# PRAME Expression in Melanoacanthomas: Expanding the Spectrum of Positive Melanocytes in Sun-Exposed Skin

**DOI:** 10.3390/dermatopathology13010014

**Published:** 2026-03-23

**Authors:** Francesco Fortarezza, Anna Poputchikova, Federica Pezzuto, Christian Ciolfi, Vincenza Guzzardo, Paolo Del Fiore, Gerardo Cazzato, Franco Bassetto, Mauro Alaibac, Angelo Paolo Dei Tos

**Affiliations:** 1 Surgical Pathology and Cytopathology Unit, University Hospital of Padova, Via Nicolò Giustiniani 2, 35128 Padova, Italy; anna.poputchikova@studenti.unipd.it (A.P.); federica.pezzuto@unipd.it (F.P.); vincenza.guzzardo@unipd.it (V.G.); angelo.deitos@unipd.it (A.P.D.T.); 2 Dermatology Unit, University Hospital of Padua, 35128 Padua, Italy; chriciolfi96@gmail.com (C.C.); mauro.alaibac@unipd.it (M.A.); 3 Soft-Tissue, Peritoneum and Melanoma Surgical Oncology Unit, Veneto Institute of Oncology IOV-IRCCS, 35128 Padova, Italy; paolo.delfiore@iov.veneto.it; 4 Pathology Unit, Department of Precision and Regenerative Medicine and Ionian Area (DiMePRe-J), University of Bari “Aldo Moro”, 70124 Bari, Italy; gerardo.cazzato@uniba.it; 5 Plastic Surgery Unit, Department of Neurosciences, University of Padua, 35128 Padova, Italy; franco.bassetto@unipd.it; 6 Department of Medicine, School of Medicine, University of Padua, 35128 Padova, Italy

**Keywords:** PRAME, melanoma, melanoacanthoma, skin tumors

## Abstract

PRAME (Preferentially Expressed Antigen in Melanoma) is increasingly used as an immunohistochemical marker in the evaluation of melanocytic lesions; however, its expression in benign melanocytic proliferations remains incompletely characterized. This study investigated PRAME expression in melanoacanthomas, with particular emphasis on its relationship with ultraviolet exposure and chronic solar damage. A consecutive series of melanoacanthomas was retrospectively analyzed. Melanocytes were identified and quantified using SOX10 immunohistochemistry, while PRAME-positive melanocytes were counted and graded semiquantitatively according to nuclear staining intensity. PRAME expression was correlated with lesion site (photoexposed versus non-photoexposed skin) and with the degree of solar elastosis. Eighty-four cases were evaluated, of which 25 (29.8%) showed at least focal PRAME positivity in melanocytes. Overall melanocytic density assessed by SOX10 did not differ significantly between photoexposed and non-photoexposed lesions. Similarly, stratification based on total PRAME-positive melanocyte counts, irrespective of staining intensity, revealed no significant association with photoexposure. In contrast, analysis restricted to melanocytes with strong nuclear PRAME expression demonstrated a significant enrichment in photoexposed lesions compared with non-photoexposed sites (*p* < 0.01). Moreover, high-intensity PRAME expression showed a positive association with increasing grades of solar elastosis. These findings indicate that strong PRAME expression in melanoacanthoma could be associated with chronic sun damage and may reflect non-specific, ultraviolet-related modulation rather than malignant transformation, underscoring the importance of contextual interpretation of PRAME immunohistochemistry in diagnostic practice.

## Simple Summary

PRAME is a marker commonly used in pathology to help distinguish malignant melanoma from benign melanocytic lesions. However, PRAME can also be detected in some benign conditions, and the reasons for this are not fully understood. In this study, we analyzed PRAME expression in melanoacanthoma, a benign skin lesion, focusing on the possible influence of sun exposure. We found that while overall PRAME positivity was relatively frequent, strong PRAME expression was more commonly observed in lesions arising on sun-exposed skin and in areas showing chronic sun damage. Importantly, this increased expression was not associated with features of malignancy. These findings suggest that strong PRAME expression in melanoacanthoma may reflect long-term ultraviolet exposure rather than malignant transformation. Our results highlight the importance of interpreting PRAME staining in the context of lesion type and sun damage to avoid diagnostic misinterpretation in dermatology and dermatopathology.

## 1. Introduction

PRAME is a cancer-testis antigen that has gained increasing relevance in surgical pathology as an immunohistochemical marker for the evaluation of melanocytic lesions [1]. Initially identified as a melanoma-associated antigen, PRAME has been progressively incorporated into routine diagnostic practice, particularly to support the distinction between benign nevi and melanoma in histologically challenging cases. Diffuse and strong nuclear PRAME expression is currently regarded as a feature favoring malignancy, especially when interpreted in conjunction with histomorphological and clinical findings.

PRAME belongs to the family of cancer-testis antigens, a group of proteins characterized by restricted expression in normal tissues and predominant localization in immune-privileged sites of the reproductive system, such as the testis [2]. In most normal somatic tissues, PRAME expression is transcriptionally silenced through promoter hypermethylation, whereas promoter hypomethylation in malignant cells leads to its re-expression. Beyond its diagnostic utility, PRAME has been implicated in key biological pathways related to tumorigenesis [3].

Despite its widespread use as a melanoma marker, the biological regulation of PRAME expression in melanocytes remains incompletely understood. While melanoma typically shows diffuse and high-level PRAME expression, focal or low-intensity PRAME positivity has been reported in a subset of benign or reactive melanocytic proliferations [4]. These observations raise important questions regarding the specificity of PRAME staining and suggest that factors other than overt malignancy may influence its expression. Epigenetic modulation, microenvironmental cues, and external stressors may all contribute to PRAME regulation, yet systematic investigations addressing these mechanisms in non-neoplastic settings are limited.

Ultraviolet (UV) radiation represents a major driver of melanocytic DNA damage and cutaneous carcinogenesis [5]. Chronic UV exposure is associated with cumulative genetic and epigenetic alterations, remodeling of the cutaneous microenvironment, and altered melanocyte–keratinocyte interactions. In this context, PRAME expression has been hypothesized to reflect, at least in part, UV-induced molecular stress or epigenetic deregulation, rather than being exclusively linked to malignant transformation [6]. However, the relationship between PRAME expression intensity, melanocytic density, and objective markers of chronic solar damage has not been systematically explored.

Melanoacanthoma is a benign keratinocytic lesion characterized by acanthosis and papillomatosis associated with an increased number of dendritic melanocytes within the epidermis. According to the most recent WHO Classification of Skin Tumours [7], “melanoacanthoma is microscopically similar to seborrhoeic keratosis or other benign squamoproliferative lesions, but it shows colonization by numerous dendritic melanocytes with bland cytological features.” In routine dermatopathological practice, a certain degree of overlap exists between melanoacanthoma and heavily pigmented seborrhoeic keratosis, and these entities are often considered to represent part of a morphological spectrum characterized by varying degrees of melanocytic colonization of an acanthotic epidermal proliferation.

Given this background, lesions displaying prominent benign melanocytic hyperplasia within keratinocytic proliferation provide a useful biological setting in which to investigate melanocyte-associated markers in a clearly non-neoplastic context. In the present study, we aim to evaluate PRAME expression in benign melanocytes colonizing acanthotic epidermal lesions and to explore its possible relationship with clinicopathological parameters, particularly chronic photodamage. We did not aim to distinguish pigmented seborrheic keratosis from melanoacanthoma diagnostically or to support a diagnostic role for PRAME in these lesions; rather, by assessing PRAME expression in this benign context, we sought to improve the understanding of PRAME positivity in non-neoplastic melanocytes and its interpretation in routine dermatopathology.

## 2. Materials and Methods

Histologically diagnosed cases of melanoacanthoma were retrospectively collected. All available hematoxylin and eosin-stained slides were reviewed by two experienced pathologists, and only acanthotic lesions with melanocytic colonization throughout the lesion, confirmed by SOX10 immunohistochemistry, were included in the study. Cases with insufficient material or equivocal morphological features were excluded. For each confirmed case, clinical data including patient age, sex, and anatomical location of the lesion were retrieved from pathology reports and clinical records. Based on lesion site, cases were subsequently classified as arising in photoexposed or non-photoexposed skin. In addition to diagnostic review, the degree of solar elastosis was assessed histologically on hematoxylin and eosin-stained sections by evaluating the amount and distribution of elastotic material within the superficial dermis adjacent to the lesion. Solar elastosis was graded semiquantitatively on a four-tier scale as follows: 0, absence of solar elastosis; 1, mild solar elastosis with scattered, individually identifiable elastotic fibers; 2, moderate solar elastosis with increased and thickened elastotic fibers; and 3, severe solar elastosis characterized by dense, amorphous aggregates of elastotic material within the dermis [7]. Immunohistochemical analyses were performed on representative formalin-fixed, paraffin-embedded tissue sections. Melanocytes were identified using SOX10 immunohistochemistry (clone EP268 (Sigma, St. Louis, MO, USA)), while PRAME expression was assessed using a monoclonal anti-PRAME antibody (clone EPR20330 (Abcam, Cambridge, UK)). The total number of melanocytes was determined by counting SOX10-positive nuclei within the lesional epidermis. PRAME-positive melanocytes were subsequently identified and counted on corresponding sections. Nuclear PRAME staining intensity was assessed semiquantitatively for each melanocyte and graded as 0 (no staining), 1+ (weak), 2+ (moderate), or 3+ (strong). Melanocyte counts were standardized to a defined epidermal area. Specifically, SOX10-positive and PRAME-positive melanocytes were counted within an epidermal area of 2 mm^2^ in each case. Counts were performed in the area showing the highest density of SOX10- and PRAME-positive melanocytes (hot spot) to ensure consistency across cases. Cell counts were independently performed by two pathologists and subsequently reviewed jointly to reach consensus. The proportion of PRAME-positive melanocytes was then calculated as the percentage of PRAME-positive cells relative to the total number of SOX10-positive melanocytes. For analytical purposes, PRAME expression was also stratified according to staining intensity, distinguishing low/moderate expression (1+/2+) from strong expression (3+). Statistical analyses were undertaken to explore the relationships among melanocytic density, PRAME expression, lesion site, and the degree of solar elastosis. Comparisons between sun-exposed and non-sun-exposed lesions were performed using Fisher’s exact test, based on the proportion of total PRAME-positive melanocytes relative to SOX10-positive melanocytes and the proportion of PRAME 3+-positive melanocytes relative to SOX10-positive melanocytes. The association between solar elastosis grade and the absolute number of SOX10-positive melanocytes, PRAME-positive melanocytes with low-to-moderate intensity (1+ and 2+), and PRAME-positive melanocytes with strong intensity (3+) was assessed, across all cases, using both Pearson’s and Spearman’s correlation coefficients. Exact *p*-values were reported throughout. All tests were two-sided, and a *p* value < 0.05 was considered statistically significant.

## 3. Results

A total of 84 consecutive cases diagnosed as melanoacanthoma were retrieved from the pathology archives and included in the study. All cases underwent PRAME immunohistochemistry. Among them, 25 cases (29.8%) showed at least focal and/or weak PRAME positivity in melanocytes and constituted the focus of the subsequent analyses. The clinical and pathological characteristics of these 25 PRAME-positive cases are summarized in Table 1. The study population showed a marked male predominance, with 80% of patients being male and 20% female. The median age at diagnosis was 63 years, with an interquartile range of 59–69 years. With regard to anatomical distribution, lesions were most frequently located in the head and neck region (60%), followed by the limbs (16%), dorsal region (12%), abdomen (8%), and breast (4%). Based on lesion site, 64% of cases arose in photoexposed skin, whereas 36% were located in non-photoexposed areas. At least one degree of solar elastosis was present in two thirds of cases.

**Figure 1:**
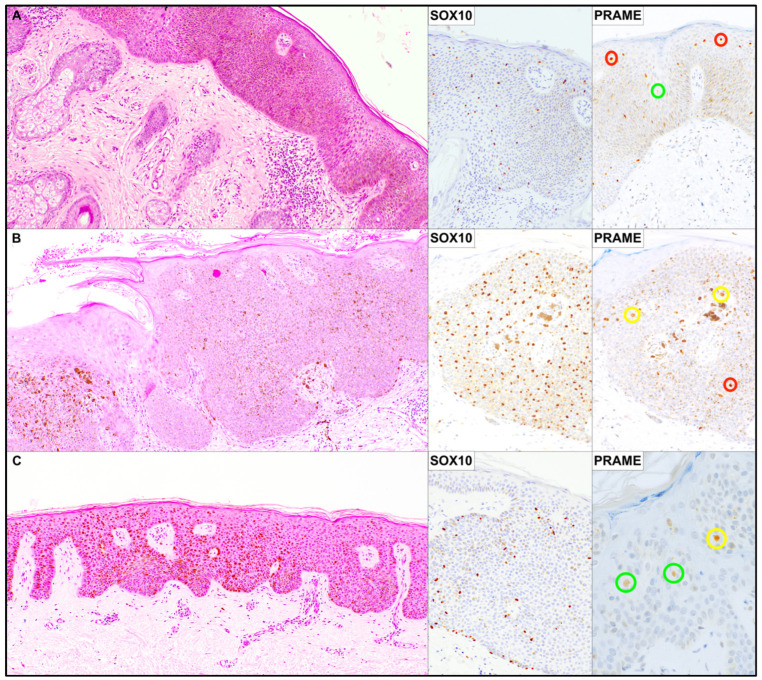
Representative illustration of three cases from the series with the corresponding immunohistochemical findings. (**A**) H&E stain, ×50; SOX10 and PRAME immunoperoxidase stains, ×100. (**B**) H&E stain, ×50; SOX10 and PRAME immunoperoxidase stains, ×100. (**C**) H&E stain, ×50; SOX10 immunoperoxidase stain, ×100; PRAME immunoperoxidase stain, ×200. Colored circles highlight nuclei according to PRAME staining intensity: green, weak expression (1+); yellow, moderate expression (2+); red, strong expression (3+).

SOX10-positive melanocyte counts ranged from 50 to 584 cells per case. PRAME-positive melanocytes with low to moderate nuclear staining intensity (1+–2+) ranged from 3 to 343 cells per case, whereas melanocytes showing strong PRAME nuclear expression (3+) ranged from 6 to 89 cells per case. The absolute number of PRAME positive melanocytes varied widely among cases and did not parallel the overall density of SOX10-positive melanocytes. Similarly, strong PRAME nuclear staining (3+) was observed only in a subset of melanocytes within each lesion, with considerable variability in the number of high-intensity PRAME-positive cells across cases. Illustrative histological and immunohistochemical images are shown in Figure 1.

**Figure 2:**
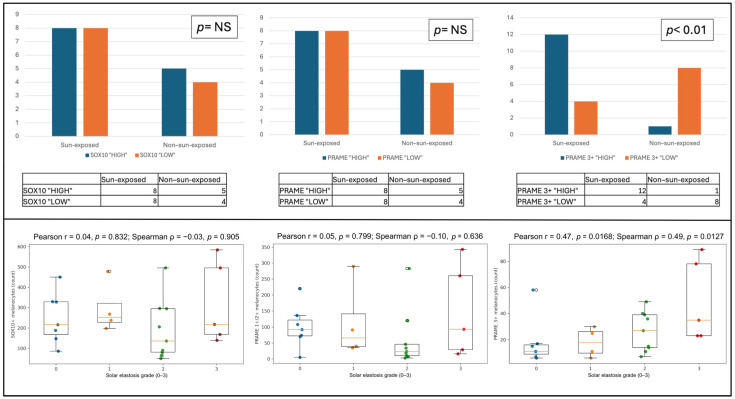
Distribution of melanocytic density, PRAME expression, and correlation with photo exposition and solar elastosis. Upper panels: distribution of cases according to lesion site (sun-exposed versus non-sun-exposed skin). Left panel: cases classified as SOX10 “HIGH” or “LOW” according to total melanocytic density assessed by SOX10 immunohistochemistry, showing no significant association with lesion site. Middle panel: cases classified as PRAME “HIGH” or “LOW” according to the PRAME/SOX10 ratio, reflecting the proportion of PRAME-positive melanocytes irrespective of staining intensity, also showing no significant association with lesion site. Right panel: cases classified as PRAME 3+ “HIGH” or “LOW” according to the PRAME 3+/SOX10 ratio, reflecting the proportion of melanocytes with strong nuclear PRAME expression; a significant enrichment of PRAME 3+ “HIGH” cases was observed in lesions arising on sun-exposed skin (*p* < 0.01). Lower panels: box plots showing the relationship between solar elastosis grade (0–3) and the absolute number, across all cases, of SOX10-positive melanocytes (**left**), low-intensity PRAME-positive melanocytes (1+/2+) (**middle**), and high-intensity PRAME-positive melanocytes (3+) (**right**). No significant correlation was observed for SOX10-positive melanocytes or low-intensity PRAME-positive melanocytes, whereas high-intensity PRAME expression showed a moderate positive correlation with increasing solar elastosis.

To evaluate whether overall melanocytic density differed according to lesion site, we compared the number of SOX10-positive melanocytes between melanoacanthomas arising in photoexposed and non-photoexposed skin. For this purpose, cases were stratified into two groups based on the median value of SOX10-positive melanocyte counts: cases with counts equal or above the median were classified as SOX10 “HIGH”, whereas those with counts below the median were classified as SOX10 “LOW”. Among lesions arising in photoexposed sites, a higher proportion of cases fell into the SOX10 “HIGH” category. In contrast, melanoacanthomas located in non-photoexposed skin showed a more balanced distribution, with 5 cases classified as SOX10 “HIGH” and 4 cases as SOX10 “LOW”. However, this difference did not reach statistical significance (Figure 2, left panel). PRAME expression was subsequently evaluated as the percentage of PRAME-positive melanocytes in each lesion, expressed as the PRAME/SOX10 ratio, irrespective of staining intensity. Cases were stratified according to the median value of total PRAME-positive melanocyte counts into two groups: PRAME “HIGH”, including cases with counts above the median, and PRAME “LOW”, including cases with counts below the median. When the distribution of PRAME “HIGH” and PRAME “LOW” cases was compared between photoexposed and non-photoexposed sites, no significant differences were observed. Both categories showed a comparable distribution across lesion sites, and statistical analysis did not reveal a significant association between overall PRAME positivity and photoexposure status (Figure 2, middle panel). Unlike the previous analyses, PRAME expression was subsequently assessed by focusing exclusively on melanocytes with strong nuclear staining, calculated as the PRAME 3+/SOX10 ratio, since this pattern is considered the most diagnostically relevant. Cases were stratified according to the median number of PRAME 3+-positive melanocytes into PRAME 3+ “HIGH” and PRAME 3+ “LOW” categories. A marked difference emerged when PRAME 3+ expression was analyzed in relation to lesion site. Among melanoacanthomas arising in photoexposed skin, the majority of cases were classified as PRAME 3+ “HIGH”, whereas only 4 cases fell into the PRAME 3+ “LOW” category. In contrast, lesions located in non-photoexposed sites showed an opposite distribution, with only 1 case classified as PRAME 3+ “HIGH” and 8 cases as PRAME 3+ “LOW”. This difference was statistically significant (*p* < 0.01), indicating a strong association between high-intensity PRAME expression and photoexposed anatomical sites (Figure 2, right panel).

The relationship between chronic solar damage and immunohistochemical findings was further explored across the entire case series by assessing the correlation between the degree of solar elastosis, used as a surrogate marker of cumulative ultraviolet exposure, and three variables: the total number of SOX10-positive melanocytes, the number of PRAME-positive melanocytes with low-intensity nuclear staining (1+–2+), and the number of PRAME-positive melanocytes with high-intensity nuclear staining (3+) (Figure 2, lower panel). No significant correlation was observed between the degree of solar elastosis and the total number of SOX10-positive melanocytes (r = 0.04), indicating that overall melanocytic density did not increase with progressive actinic damage. Similarly, low-intensity PRAME expression (1+–2+) did not show a meaningful association with solar elastosis (r = 0.05), with a wide dispersion of values across all elastosis grades and no evident trend toward higher counts at increasing levels of solar damage. In contrast, analysis of high-intensity PRAME expression revealed a moderate positive correlation with the degree of solar elastosis (r = 0.47). Higher numbers of PRAME 3+-positive melanocytes were preferentially observed in lesions displaying moderate to severe solar elastosis (grades 2–3), whereas lower counts predominated in cases with absent or mild elastosis. These findings indicate that strong nuclear PRAME expression is selectively associated with chronic sun-damaged skin.

## 4. Discussion

The diagnostic utility of PRAME immunohistochemistry in melanocytic pathology has been widely investigated in recent years. Initially identified as a tumor-associated antigen expressed in melanoma and later demonstrated to be detectable by immunohistochemistry in formalin-fixed, paraffin-embedded tissue [8], PRAME has emerged as a valuable ancillary marker in the assessment of diagnostically challenging melanocytic proliferations. Subsequent diagnostic accuracy studies have further refined its interpretative framework by proposing quantitative thresholds to support clinical use. In a STARD-compliant study, the proportion of PRAME-positive melanocytes was shown to be significantly higher in melanomas than in benign lesions, with a PRAME score exceeding 75% demonstrating high specificity for melanoma [9]. PRAME immunohistochemistry has also proved helpful in specific diagnostic settings, such as melanoma arising in association with a pre-existing nevus, where strong and diffuse PRAME expression is typically confined to the malignant component, while the adjacent benign nevus shows absent or only focal staining [10]. Likewise, PRAME has been shown to be useful in the evaluation of lentigo maligna, both for diagnostic purposes and for margin assessment [11]. More recently, attempts have been made to standardize and objectify PRAME evaluation through digital pathology, including a quantitative method combining PRAME and SOX10 staining to generate a PRAME index, with diagnostic performance comparable to manual qualitative scoring but with greater reproducibility and reduced operator dependency [12]. Overall, the available literature supports the role of PRAME as a useful ancillary tool in the differential diagnosis between benign and malignant melanocytic proliferations. At the same time, these studies underscore that PRAME should not be interpreted in isolation, as staining patterns may be influenced by technical variables, scoring criteria, and biological context, and must therefore always be integrated with morphological and clinical findings.

Despite its recognized diagnostic value, PRAME immunohistochemistry is not without pitfalls. In a large institutional series, Turner et al. reported diffuse PRAME expression in approximately 80% of conventional melanomas but also documented positivity in a subset of dysplastic nevi and in some diagnostically ambiguous melanocytic proliferations. Occasional PRAME expression was also identified in certain non-melanocytic malignancies [13]. In this context, evaluating PRAME expression in unequivocally benign melanocytic settings may offer further insight into the biological variability of PRAME staining and help refine awareness of its diagnostic limitations.

The first relevant observation of our study is that focal PRAME positivity is not uncommon in melanoacanthoma, being detected in approximately 30% of cases. Importantly, simple PRAME positivity—particularly when of low intensity—showed no significant association with lesion site, melanocytic density, or degree of solar elastosis. Neither the total number of SOX10-positive melanocytes nor the overall number of PRAME-positive melanocytes differed significantly between photoexposed and non-photoexposed lesions. These findings indicate that melanocytic hyperplasia per se, as well as low-level PRAME expression, are not directly influenced by chronic ultraviolet exposure and should not be overinterpreted as indicators of malignant transformation.

In contrast, a distinct pattern emerged when PRAME expression was analyzed by restricting the evaluation to melanocytes with strong nuclear staining. High-intensity PRAME expression was significantly enriched in lesions arising in photoexposed skin and showed a moderate positive correlation with the degree of solar elastosis. Notably, this association was not observed for SOX10-positive melanocyte counts or for PRAME low-intensity-positive cells, underscoring that staining intensity, rather than mere PRAME positivity, represents the critical discriminating variable. These results suggest that strong nuclear PRAME expression is selectively associated with chronically sun-damaged skin, even in the absence of malignancy.

Our findings are consistent with previous studies on PRAME expression in sun-damaged skin. Olds et al. [6] reported that benign sun-exposed lesions may show sparse PRAME-positive melanocytes, whereas melanoma in situ typically displays much higher densities, leading to the proposed cutoff of ≥10 PRAME-positive melanocytes per millimeter. However, this threshold cannot be directly applied to melanoacanthoma or other pigmented keratoses, as these lesions are variably acanthotic and therefore not meaningfully comparable on a per-millimeter epidermal length basis. In this setting, our study is relevant not because it supports that cutoff, but because it further indicates that chronic photo exposure may itself induce PRAME expression in benign melanocytes. This point should be taken into account when interpreting PRAME immunohistochemistry in routine practice, particularly to avoid overcalling limited PRAME positivity in sun-damaged lesions.

From a biological perspective, these findings are consistent with the known epigenetic regulation of PRAME and its interaction with ultraviolet-induced skin damage. As a cancer-testis antigen, PRAME is transcriptionally silenced in most normal somatic tissues through promoter hypermethylation, whereas promoter hypomethylation leads to its re-expression in malignant cells. Chronic ultraviolet exposure is known to induce cumulative genetic and epigenetic alterations in the skin, including changes in DNA methylation patterns, oxidative stress, and remodeling of the epidermal and dermal microenvironment [14]. In this setting, PRAME expression may reflect UV-driven epigenetic deregulation rather than an intrinsic neoplastic program.

The interaction between ultraviolet exposure, retinoic acid (RA) signaling, and PRAME expression provides a further biologically plausible framework to interpret our findings. Retinoic acid plays a central role in epidermal homeostasis, regulating keratinocyte and melanocyte differentiation, proliferation, and apoptosis through binding to nuclear retinoic acid receptors (RARs) [15]. Chronic UV exposure interferes with RA metabolism and signaling at multiple levels, resulting in a functional “retinoid-deficient” state in photo-damaged skin [16]. PRAME, acting as a dominant repressor of RAR signaling, may further amplify this effect. The selective enrichment of PRAME 3+ melanocytes in lesions with marked solar elastosis suggests that PRAME activation may represent an adaptive or stress-related response within a UV-altered microenvironment where RA signaling is already compromised.

These observations have direct implications for diagnostic practice. PRAME immunohistochemistry has rapidly become a widely used adjunct in the evaluation of melanocytic lesions, and diffuse strong nuclear staining remains a robust feature supporting melanoma. However, our data reinforce the need for a contextual, quantitative, and intensity-based interpretation of PRAME staining. Focal PRAME positivity or limited numbers of PRAME 3+ melanocytes, particularly in sun-exposed skin and in lesions with benign morphology, should not be equated with malignancy in the absence of corroborating histological and clinical features.

This study has several limitations that should be acknowledged. First, its retrospective design may introduce inherent selection biases related to case retrieval and available tissue material. In addition, the selection of melanoacanthoma cases may have included lesions showing partial morphological overlap with pigmented seborrheic keratoses. Indeed, the boundary between these entities can be subtle, and they are often considered part of a spectrum of keratinocytic proliferations with varying degrees of melanocytic colonization. However, the primary aim of this study was not to establish a strict diagnostic distinction between these lesions, but rather to investigate PRAME expression in benign melanocytes colonizing acanthotic epidermal proliferations, providing a biological setting to explore PRAME positivity in non-neoplastic melanocytes. Additional potential confounders should also be considered, including anatomical site, age-related actinic changes, and technical variability in PRAME staining intensity. Finally, the relatively limited sample size may restrict the strength of statistical correlations, and larger studies will be necessary to further validate the observed associations.

## 5. Conclusions

In conclusion, our study expands the spectrum of benign melanocytic settings in which PRAME expression may be encountered and demonstrates that strong nuclear PRAME expression can be associated with chronic sun damage in the absence of malignancy. When interpreted considering quantitative thresholds and distribution patterns proposed in the literature, PRAME remains a valuable diagnostic marker. However, our results confirm an important clinical implication, namely that PRAME positivity is not exclusively restricted to neoplastic melanocytes and may also be observed in benign lesions. These findings highlight the importance of interpreting PRAME immunohistochemistry in conjunction with staining intensity, extent, morphological context, and environmental factors to avoid overdiagnosis and to refine the interpretative framework of PRAME immunohistochemistry in the evaluation of diagnostically challenging melanocytic lesions in routine practice.

## Figures and Tables

**Table 1:** Clinical–pathological features of the cohort.

	Sex	Age	Site	Photoexposure	Solar Elastosis (0–3)	% PRAME/SOX10	% PRAME 3+/SOX10
1	M	77	Scalp	Yes	2	67.10%	9.90%
2	M	86	Temple	Yes	1	30.30%	12.60%
3	F	84	Dorsal	No	1	61.90%	1.30%
4	M	67	Cheek	Yes	3	74.00%	15.20%
5	M	65	Cheek	Yes	3	68.30%	15.80%
6	M	35	Thigh	No	0	44.70%	3.30%
7	F	60	Breast	No	0	89.50%	8.10%
8	F	59	Lumbar	No	0	58.00%	9.00%
9	M	68	Flank	No	1	38.10%	4.10%
10	M	78	Scapula	No	0	37.00%	2.80%
11	M	66	Clavicle	Yes	2	65.40%	8.60%
12	M	63	Forehead	Yes	2	49.70%	9.10%
13	F	33	Abdomen	No	0	51.30%	2.40%
14	M	69	Temple	Yes	1	29.40%	12.60%
15	M	52	Shoulder	Yes	2	21.50%	16.90%
16	M	63	Flank	No	0	37.60%	4.60%
17	M	60	Scalp	Yes	3	53.20%	10.60%
18	F	60	Face	Yes	2	46.30%	29.40%
19	M	61	Temple	Yes	2	64.00%	28.00%
20	M	57	Forehead	Yes	2	24.40%	16.70%
21	M	64	Temple	Yes	3	22.30%	25.20%
22	M	70	Scalp	Yes	2	23.80%	12.20%
23	M	71	Vertex	Yes	2	23.80%	18.90%
24	M	48	Dorsal	No	0	42.90%	39.50%
25	M	45	Scalp	Yes	3	31.00%	13.70%

## Data Availability

The original contributions presented in this study are included in the article. Further inquiries can be directed to the corresponding author.

## Abbreviations

The following abbreviations are used in this manuscript:

PRAME	Preferentially Expressed Antigen in Melanoma
